# The mitochondria as a potential therapeutic target in cerebral I/R injury

**DOI:** 10.3389/fnins.2024.1500647

**Published:** 2025-01-07

**Authors:** Susu Fang, Wenzhou Huang, Xinhui Qu, Wen Chai

**Affiliations:** ^1^The Second Department of Neurology, Jiangxi Provincial People’s Hospital and The First Affiliated Hospital of Nanchang Medical College, Nanchang, Jiangxi, China; ^2^Institute of Geriatrics, Jiangxi Provincial People’s Hospital, The First Affiliated Hospital of Nanchang Medical College, Nanchang, Jiangxi, China; ^3^Department of Orthopedics, The Second Affiliated Hospital, Jiangxi Medical College, Nanchang University, Nanchang, Jiangxi, China; ^4^Jiangxi Provincial Key Laboratory of Spine and Spinal Cord Disease, Nanchang, Jiangxi, China; ^5^Department of Neurology, Jiangxi Provincial People’s Hospital, The First Affiliated Hospital of Nanchang Medical College, Nanchang, Jiangxi, China

**Keywords:** cerebral ischemia/reperfusion injury (CIRI), mitochondrion, acetylation, Ca^2+^, ROS, inflammation, mitochondrial turnover

## Abstract

Ischemic stroke is a major cause of mortality and disability worldwide. Among patients with ischemic stroke, the primary treatment goal is to reduce acute cerebral ischemic injury and limit the infarct size in a timely manner by ensuring effective cerebral reperfusion through the administration of either intravenous thrombolysis or endovascular therapy. However, reperfusion can induce neuronal death, known as cerebral reperfusion injury, for which effective therapies are lacking. Accumulating data supports a paradigm whereby cerebral ischemia/reperfusion (I/R) injury is coupled with impaired mitochondrial function, contributing to the pathogenesis of ischemic stroke. Herein, we review recent evidence demonstrating a heterogeneous mitochondrial response following cerebral I/R injury, placing a specific focus on mitochondrial protein modifications, reactive oxygen species, calcium (Ca^2+^), inflammation, and quality control under experimental conditions using animal models.

## 1 Overview of the mitochondrial function in acute cerebral ischemia/reperfusion injury

The mitochondria are double-membrane organelles with distinct genomes, known as the mitochondrial DNA, (mtDNA), which encodes 13 proteins. The proteins encoded by the mtDNA participate in electron transport during oxidative phosphorylation (OXPHOS). High-energy electrons from NADH and FADH_2_ produced by substrate metabolism in the Krebs cycle pass via the electron transport chain (ETC) to oxygen. Meanwhile, the ETC transports protons (released from oxidized NADH and FADH_2_) from the mitochondrial inner membrane to the intermembrane, thus generating a proton motive force (pmf), as the inner membrane is impermeable to H^+^ ions. The PMF is composed of a mitochondrial membrane potential and a small pH gradient, which together drive ATP synthesis (Berry et al., 2018). Mitochondria can store mM Ca^2+^ and is tightly controlled by ion channels and transporters localized in the inner membrane (Clapham, 2007; Garbincius and Elrod, 2022). An increase in mitochondrial Ca^2+^ activates several key enzymes related to oxidative metabolism, OXPHOS, and mitochondrial superoxide dismutase (Denton et al., 1978; McCormack and Denton, 1979; Territo et al., 2000; Hopper et al., 2006; Glancy et al., 2013). Mitochondrial Ca^2+^ overload can dissipate the mitochondrial membrane potential and slow the ETC transport rate, resulting in the generation of mitochondrial reactive oxygen species (mtROS) due to electron leakage from the ETC. To fight the overload of mitochondrial Ca^2+^, the mitochondrial permeability transition pore (mPTP) can be switched to the open state, in order to release mitochondrial Ca^2+^ into the cytosol, which may promote mitochondrial swelling, cytochrome C release, and apoptosis (Bernardi et al., 2015; Garbincius and Elrod, 2022). Injury to the mitochondria can result in the release of mitochondrial content, including mitochondrial proteins and mtDNA, which could initiate a sterile inflammatory response in the innate immune system due to the bacterial origin (Shimada et al., 2012; Eleftheriadis et al., 2016). In addition to their fundamental role in energy metabolism, mitochondria are also involved in changes in many molecular and biochemical signaling molecules, such as Ca^2+^, reactive oxygen species (ROS), acetyl-coenzyme A (Ac-CoA), nicotinamide adenine dinucleotide (NADH/NAD^+^), among many others. These mitochondrial metabolites can act as secondary messengers, defined as mitochondrial signaling, to induce significant genetic changes in neuronal activity and function (Calvo-Rodriguez et al., 2020; Cid-Castro and Morán, 2021; Sun et al., 2023).

The brain is a highly energy-demanding organ and the ATP production rate in mitochondria is proximity to 9 micromol/g/min in humans (Du et al., 2007; Sokoloff, 1960), indicating a high metabolic requirement for neural processing (Attwell and Laughlin, 2001). It is estimated that the human brain contains 100 billion neurons, which perform a wide range of integrative functions and communication (Herculano-Houzel, 2009; van den Heuvel and Sporns, 2013).

Approximately 90% of all stroke cases are classified as ischemic stroke, which can result from embolism or thrombosis (Johnson et al., 2016). Focal ischemic animal models are commonly used to study ischemic stroke clinically (Lipton, 1999). Cerebral ischemia/reperfusion (I/R) injury, a primary focal ischemia model, occurs when the blood supply to the brain is blocked and restored, resulting in several severe biochemical and metabolic disorders in neurons, selectively vulnerable to ischemia. The brain tissue at the center of the ischemic core in the parenchyma, known as the stroke region or infarction region, which has been injured by ischemia shows edema and cellular swelling with permanent necrotic cell death within minutes (Smith, 2004). If reperfusion treatment is delayed, the infarction region proceeds from the brain parenchyma (ischemic core) to the penumbra (risk zone) due to the release of pathogenic factors, including glutamate excitotoxicity, Ca^2+^ overload, oxidative stress, and inflammation (Moskowitz et al., 2010). Although neuronal death may not be necessary within the infarction region (at least in rodents), irreversible neuronal damage occurs within the second few minutes of ischemia, if cerebral reperfusion treatment is ineffective (Moskowitz et al., 2010; Sonderer and Katan Kahles, 2015). Every 5-min delay in cerebral endovascular reperfusion can cause a worse disability outcome (Sheth et al., 2015), while a 30-min delay has been associated with a 10.6% drop in the probability of positive disability outcomes (Khatri et al., 2009). Although timely reperfusion is critical for preventing long-term ischemic damage and adverse neurological outcomes, reperfusion itself can initiate secondary brain injury (Moskowitz et al., 2010). Mitochondrial dysfunction is commonly observed in cerebral I/R injury. Although it is thought to be a maladaptive feedback, the underlying mechanisms connecting mitochondrial dysfunction to the onset or progression of cerebral stroke appear to be complex, and remain only poorly understood. Accumulating data have revealed a large number of mitochondrial molecular mechanisms that contribute to the pathogenesis of cerebral stroke.

**FIGURE 1:**
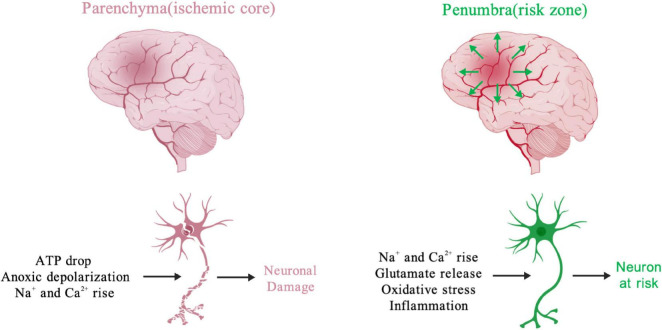
Activators of processes causing neuronal functional changes during cerebral ischemia. The early ischemic changes, including loss of ATP, ion imbalance and rising of cellular Na^+^ and Ca^2+^, promote neuronal injury and even death in parenchyma. The ischemic glutamate release from parenchyma could cause excitotoxic effect in peri-infarcted region (penumbra). Meanwhile, many neuronal risk factors, like oxidative stress, inflammation, Na^+^ and Ca^2+^ rise irreversibly damage cerebral tissue in the ischemic penumbra. Created with BioGDP.com (Jiang et al., 2024).

At the cellular level (Figure 1), the cerebral ischemic core undergoes the rapid depletion of energy and oxygen, resulting in an increase in extracellular K^+^ and neuronal cytosolic Na^+^ and Ca^2+^ within minutes (Lipton, 1999). The Ca^2+^ accumulation is thought as a trigger to initiate a series of cellular events, including mitochondria-dependent apoptotic pathway (Lipton, 1999; Broughton et al., 2009; Fricker et al., 2018). In the ischemic penumbra, blood flow is not fully blocked, instead occurring at a slow rate in the presence of glucose. Therefore, neuronal metabolism is reprogrammed to increase reliance on glycolysis from mitochondrial OXPHOS, resulting in intracellular lactic acidosis (Toth et al., 2020). Decreased tissue pH is inversely correlated with tissue lactate concentration following cerebral ischemic insult (Katsura et al., 1991). Intracellular acidosis activates the Na^+^/H^+^ ion exchange to extrude excess protons. Subsequently, the Na^+^/Ca^2+^ exchanger attempts to remove excess Na^+^, resulting in an increase in cytosolic Ca^2+^ (Kintner et al., 2004; Luo et al., 2005), triggering a mitochondrial Ca^2+^ overload, opening of the mPTP, and mitochondrial depolarization, and ultimately eliciting mitochondrial injury and dysfunction. It is believed that the excess accumulation of extracellular glutamate released from the parenchyma mediates excitotoxic synaptic transmission to facilitate Ca^2+^ influx into the ischemic penumbra, amplifying the mitochondrial injury cascade (Deb et al., 2010). As mentioned above, Ca^2+^ is a critical factor contribution to the generation of mitochondrial reactive oxygen species (mtROS), mPTP opening, mitochondrial rupture, and apoptosis. Danger signals released from injured or dying mitochondria further contribute to the inflammatory responses after ischemia (Iadecola and Anrather, 2011). Acute cerebral reperfusion can induce a burst of oxidative stress generated by the re-energization of mitochondria, which further exacerbates ischemic injury (Piantadosi and Zhang, 1996; Chouchani et al., 2014). Importantly, many factors related to mitochondrial function, including mitochondrial lipid oxidation, respiratory deficiency, metabolic crisis, and calcium overload, converge on excessive ROS, making the latter a critical target for protection against cerebral I/R (Kalogeris et al., 2014; Chouchani et al., 2016). These compounded effects induced by cerebral ischemia together with reperfusion contribute to mitochondrial dysfunction and neuronal demise, and have been shown to contribute to the final infarction size.

**FIGURE 2:**
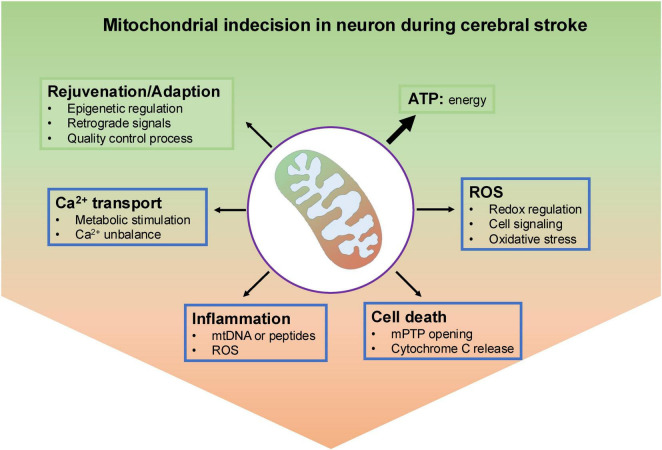
An overview of mitochondrial indecision in neuron under ischemic insult. Under normal conditions, neuronal mitochondria produce most ATP through oxidative metabolism. To keep a population of healthy mitochondria, they have several effective and well-organized mechanisms to remove dysfunctional parts, like epigenetic regulation, mitochondrial-nuclear communications and mitochondrial quality control process. In addition, mitochondrial reactive oxygen species generated by electron transport chain (ETC) could either act as molecular signals or danger stressor to damage mitochondria. Also, mitochondria calcium served as a double-edged sword, either activates mitochondrial metabolism-related enzymes or results in the opening of the mitochondrial permeability transition pore (mPTP) and ultimately neuronal cell death. Leak of mitochondrial contents including DNA, proteins, RNA and mtROS cloud cause inflammation.

In this study, we summarize the recent advances in this field, placing an emphasis on the pathophysiological regulation of mitochondrial dysfunction and the impact of the interplay between mitochondrial function and neuronal death after cerebral I/R injury (Figure 2).

## 2 Mitochondrial protein acetylation in cerebral I/R injury

Post-translational modifications are commonly involved in neuronal activity, among which acetylation is one of the most studied modifications in the pathophysiology of cerebral injury (Klimova et al., 2018). Protein acetylation can be mediated either by acetyltransferases to transfer an acetyl group, or by a reverse reaction performed by deacetylases. Within the mitochondria, acetyl-CoA is the only donor of the acetyl group for acetylation, whereas NAD^+^, a substrate for deacetylases, removes the acetyl group from target proteins (Choudhary et al., 2014). Thus, changes in acetyl-CoA and NAD^+^ levels are closely associated with mitochondrial function and the development of cerebral stroke.

Acetyl-CoA is primarily generated in the mitochondrial matrix from various energy substrates, and can be synthesized into N-acetylaspartate (NAA) in neurons. NAA is thought to act as a storage substance for acetyl-CoA, while its level decreases in neurons during cerebral I/R (Demougeot et al., 2003; Bittsansky et al., 2012; Igarashi et al., 2015). Although the pathophysiological roles of NAA in neurons remain poorly understood, these findings indirectly indicate changes in acetyl-CoA levels during neuronal damage. GCN5L1 and Sirt3 are mitochondrial acetyltransferases and deacetylases, respectively (Lombard et al., 2007; Wu K. et al., 2021). Deletion of GCN5L1 in mice was shown to reduce mitochondrial protein acetylation, whereas its overexpression exerts the opposite effect (Wang et al., 2017; Thapa et al., 2018). GCN5L1 is important for controlling mitochondrial biogenesis and mitophagy, indicating that the role of GCN5L1 may involve maintenance of a functional population of healthy mitochondria in neurons (Webster et al., 2013; Scott et al., 2014). Downregulation of Sirt3 has been observed during cerebral I/R injury (Zhao et al., 2018; Yang Y. et al., 2021), while ablation of Sirt3 was associated with increased neuronal death and neurological deficits following brain ischemia (Yang Y. et al., 2021). In contrast, the overexpression of Sirt3 *in vivo* reduced the infarction size and played a protective role in ischemic stroke (Xiaowei et al., 2023). A number of neuroprotective mitochondrial processes are regulated by Sirt3 in neuroprotection, such as mPTP opening, mitochondrial unfolded protein response, mitochondrial dynamics, and mitophagy (Zhao et al., 2018; Yang Y. et al., 2021; Wei et al., 2023; Xiaowei et al., 2023). Supporting this notion, several mitochondrial proteins, including superoxide dismutase (SOD2), mitochondrial fusion protein (OPA1), and Cyclophilin D (CypD), function as direct targets of Sirt3 deacetylase activity (Hafner et al., 2010; Samant et al., 2014; Traba et al., 2015). Indeed, recent studies have shown that NAD^+^ levels are depleted in brain tissue following an ischemic insult, and that NAD^+^ supplementation is beneficial in reducing cerebral infarct volume through Sirt3-dependent mechanisms (Klimova et al., 2020; Wang X. X. et al., 2023). Likely, the deacetylation regulated by Sirt3 is critical for protecting mitochondrial function and preventing neuronal death in ischemic stroke. However, a large gap remains in our understanding of how Sirt3 uses NAD^+^ to deacetylate specific proteins, resulting to biological consequences. In addition to its bioavailability in Sirt3, NAD^+^ is involved in a broad range of cellular functions, including energy metabolism, inflammation, redox status, calcium homeostasis, and DNA repair (Rajman et al., 2018; Zapata-Perez et al., 2021). As such, the role of NAD^+^ in neuroprotection requires further evaluation.

## 3 Mitochondrial Ca^2+^ in cerebral I/R injury

Ca^2+^ homeostasis has been recognized as a critical process in neurons, which is involved in multiple neuronal functions, including neurotransmitter release, excitability, neurite outgrowth, synaptic plasticity, gene transcription, and cell survival (Wang and Michaelis, 2010). During ischemic stroke, glutamate as a major excitatory neurotransmitter activates N-methyl D-aspartate receptors (NMDARs), which could trigger an excessive Ca^2+^ accumulation and subsequent death in neurons (Moskowitz et al., 2010). Because neuronal mitochondria are in proximity to the plasma membrane, and physically interact with the endoplasmic reticulum (ER), it is thought that mitochondria act as Ca^2+^ sinks under ischemic conditions, while the resultant Ca^2+^ overload contributes to mitochondrial dysfunction (Schafer et al., 2014). Cerebral ischemia induces cytosolic Ca^2+^ influx through nonselective cation and Ca^2+^ channels, promoting mitochondrial Ca^2+^ accumulation in a synchronized manner (De Stefani and Rizzuto, 2014; Kumar et al., 2014). In support of these findings, inhibition of mitochondrial Ca^2+^ uptake could protect from ischemic neuronal cell death (Novorolsky et al., 2020; Nichols et al., 2018). In contrast, mitochondrial Ca^2+^ can activate three enzymes in the Krebs cycle (pyruvate dehydrogenase, isocitrate dehydrogenase, and α-ketoglutarate dehydrogenase) to regulate mitochondrial metabolism via phosphorylation modification (Hopper et al., 2006). It has also been proposed that mitochondrial Ca^2+^ regulates the expression of other mitochondrial proteins associated with OXPHOS, ROS, and mPTP opening (Zhou and Tian, 2018). These findings indicate that mitochondrial Ca^2+^ is essential for basal mitochondrial function, and plays a pathological role in neurotoxicity.

The exact quantity of Ca^2+^ that must be taken up by the mitochondria before reaching a turning point resulting in a neurotoxic level in cerebral ischemia and reperfusion is unclear. Most studies have demonstrated that mitochondrial Ca^2+^ accumulation and related disturbances in neuronal function could be derived from experimental data using genetic approaches. For example, the mitochondria-associated ER membrane (MAM) protein, glucose-regulated protein 75 (GRP75), bridges the physical connection between the ER and mitochondria and facilitates Ca^2+^ import into the mitochondria (Szabadkai et al., 2006). GRP75 expression is positively correlated with neuronal mitochondrial Ca^2+^ (Szabadkai et al., 2006; Honrath et al., 2017; Liang et al., 2021), but the neuronal biological outcomes following an ischemic insult remain controversial (Xu et al., 2009; Wen et al., 2022). Indeed, cytosolic Ca^2+^ overload has been observed during cerebral ischemia, propagating the Ca^2+^ signal and potentially impairing mitochondrial function owing to the ectopic accumulation of mtROS and/or cell death. The opening of the mPTP and mitochondrial oxidative stress caused by cytosolic Ca^2+^ overload may also play a mechanistic role in mitochondrial membrane potential collapse, OXPHOS uncoupling, ATP production deficiency, and necrosis in neurons.

Mitochondrial Ca^2+^ is predominantly balanced by the mitochondrial calcium uniporter (MCU) complex for influx, and the sodium/calcium exchanger (NCLX) for efflux (Palty et al., 2010; Baughman et al., 2011; De Stefani et al., 2011). Research in mouse heart models has provided evidence that the MCU is dispensable for both basal and pathological cardiac functions, which could occur as a result of a compensatory adaptations following MCU inactivation during development and aging (Pan et al., 2013; Murphy et al., 2014; Holmstrom et al., 2015; Garbincius et al., 2020; Liu, 2020). MCU is required for rapid Ca^2+^ uptake, while the acute deletion of MCU (tamoxifen-inducible mouse knockout model) in the heart blocks acute mitochondrial Ca^2+^ uptake and MPTP opening, and protects against myocardial I/R injury (Kwong et al., 2015). These findings suggest that the role of the MCU may be most pathologically relevant in cellular death caused by mitochondrial Ca^2+^ overload, such as in the cerebral or myocardial ischemia-reperfusion injury. However, we observed that the inhibition of MCU by dominant negative overexpression in the heart affects cytoplasmic Ca^2+^ homeostasis and cardiomyocyte death, in response to ischemia-reperfusion injury potentially by Bax-dependent pathway (Rasmussen et al., 2015), suggesting that the role of MCU is not limited to rapid Ca^2+^ uptake in mitochondria, but also may involve in extramitochondrial pathway. These cardiac studies could explain why the role of the uniport in cerebral ischemic injury is controversial; indeed, different manipulation approaches of MCU inhibition have yielded disparate data both supporting and refuting protection from cerebral ischemic insult in animal models (Zhao et al., 2013; Nichols et al., 2017; Nichols et al., 2018; Qin et al., 2023). Compared with MCU, the efflux rate of NCLX is slower, and limits the overall mitochondrial flux, increasing mitochondrial susceptibility to stress (Rudolf et al., 2004). The role of NCLX in the ischemic brain remains poorly documented, although it is considered to be largely involved in cognitive performance, although findings in animal studies remain controversial (Stavsky et al., 2021; Cabral-Costa et al., 2023; Jadiya et al., 2023). In support of the mitochondrial Ca^2+^ overload hypothesis, increased NCLX activity via phosphorylation improves neuronal survival in response to excitotoxic insult, and protects against cognitive decline (Rozenfeld et al., 2022). In addition, the conditional knockout of NCLX in the mouse heart results in mitochondrial calcium overload and cardiac death. Overexpression of NCLX in the mouse heart attenuates the cell death caused by ischemia-reperfusion injury, and protects against pathological remodeling after myocardial infarction (Luongo et al., 2017). These observations confirm that NCLX is critical for controlling mitochondrial Ca^2+^ levels, and reinforce the notion that mitochondrial Ca^2+^ overload is detrimental (Luongo et al., 2017). To date, the pathophysiological significance of NCLX in cerebral ischemia-reperfusion injury has not been fully elucidated.

## 4 Mitochondrial dysfunction and Inflammation in cerebral I/R injury

Cerebral I/R injury is concomitant with an inflammatory response, as observed by elevated levels of circulating inflammatory cytokines in patients and animals (Ferrarese et al., 1999; Offner et al., 2006; Chapman et al., 2009; Iadecola and Anrather, 2011). However, clinical trials to neutralize elevated proinflammatory cytokines have thus far failed to induce any protection in reducing infarct size (Emsley et al., 2005; Smith et al., 2018). As such, a better understanding of the mechanisms underlying the inflammatory response and cerebral I/R injury is essential for developing a therapeutic strategy for ischemic stroke. Inflammation associated with ischemic stroke is activated by endogenous molecules, but not foreign stimuli, indicating a state of activated innate immunity (Iadecola and Anrather, 2011). The release of endogenous molecules, also known as damage-associated molecular patterns (DAMPs), is recognized by specific pattern recognition receptors (PRRs) in the innate immune system (Matzinger, 1994). Mitochondrial DAMPs have at least two unique molecular characteristics, and are evolutionary endosymbionts derived from bacteria: mtDNA rich in unmethylated CpG motifs and N-formyl peptides. Accumulating data has suggested that mtDNA and N-formyl peptides act on TLR9 and formyl peptide receptors, respectively, to activate inflammatory responses in various cell types, especially immune cells (Zhang et al., 2010; Wenceslau et al., 2014; Wenceslau et al., 2015; West and Shadel, 2017). Moreover, cytosolic mtDNA, irrespective of its bacterial characteristics, could be recognized by cyclic GMP-AMP synthase (cGAS) and activate the adaptor protein stimulator of interferon genes (STING) (Rongvaux et al., 2014; Yu et al., 2020). The activation of cGAS/ STING pathway mediates the inflammatory response associated with the production of type I interferons (IFNs) and proinflammatory cytokines (Kim et al., 2023). In addition to these two molecular patterns, other mitochondria-derived damage-associated molecular patterns, such as oxidized phospholipids, Ca^2+^, ATP, and RNA, have also been shown to induce inflammatory response (Gorini et al., 2013; Iyer et al., 2013; Mitoma et al., 2013; Horng, 2014; Eleftheriadis et al., 2016). One important step in the activation of innate immunity is the assembly of the NLRP3 inflammasome, which activates cysteine protease caspase-1. The activation of caspase-1 triggers the cleavage of the proinflammatory cytokines pro-interleukin-1β (pro-IL-1β) and pro-IL-18, ultimately resulting in cytokine release and cell death (Kanneganti, 2015). Multiple studies have implicated mitochondrial activities are involved in the assembly and activation of NLRP3. For example, mtROS itself could act as a trigger or induce mtDNA oxidative lesions and modulate NLRP3 activation (Nakahira et al., 2011; Zhou et al., 2011; Traba et al., 2015). A study reported that NLRP3 inflammasome activation is dependent on the level of cellular NAD^+^ which regulates α-tubulin acetylation. Acetylated α-tubulin mediates the apposition of mitochondrial ASC (apoptosis associated speck-like protein containing a CARD, adapt protein of NLRP3) to NLRP3 on the endoplasmic reticulum, subsequently activating the inflammasome (Misawa et al., 2013).

Elevated levels of circulating mtDNA and NLRP3 have been observed at an early time point after ischemia in animals, as in stroke patients, indicating a close relationship and early immune activation during ischemic stroke (Tsai et al., 2011; Franke et al., 2021; Wang Y. et al., 2021; Bellut et al., 2023). In support of this concept, the inhibition of NLRP3 mitigates neuroinflammation and leads to better outcomes, whereas the addition of mtDNA displays the opposite effect after I/R injury (Fann et al., 2013; Yang et al., 2014; Franke et al., 2021; Kong et al., 2022; Bellut et al., 2023). Circulating mitochondrial N-formyl peptides have been reported to increase and correlate with the magnitude of brain edema in patients with intracerebral hemorrhage; however, there is little evidence in cases of I/R injury (Li et al., 2021). Furthermore, recent study suggested that activation of microglial cGAS/STING axis after cerebral I/R was associated with release of mtDNA, and block of this pathway could alleviate I/R-induced neuroinflammation and brain injury (Liao et al., 2020). As such, the inflammatory response blockade induced by mitochondrial dysfunction could be an attractive treatment target, or at least could extend the therapeutic time window for ischemic stroke in humans.

## 5 Mitochondrial ROS in cerebral I/R injury

ROS are oxygen-based chemical species that consist of superoxides, hydroxyl radicals, and hydrogen peroxide. Owing to their highly reactive activities, ROS not only increases cellular oxidative stress, but also triggers cellular signaling events. The transient generation of ROS within restrictions appears to be adaptive, beneficial, and compensatory. For example, ischemic preconditioning (IPC) provides neuronal protection following I/R injury (Puisieux et al., 2004; Liu J. et al., 2005). Excessive ROS production is thought to be detrimental to cellular activity, and has been attributed to excessive ROS generation that outstrips the capacity of endogenous antioxidant systems. Given that quantifying the exact amount of ROS and distinguishing the concentration range from cellular benefits (homeostatic and adaptive) to impaired function (reversible and irreversible, even death) is difficult, scavenging ROS either by antioxidant or genetic manipulation could be an effective method to address this consideration (Li S. et al., 2023).

The production of mtROS occurs at least at 11 sites, either in the mitochondrial inner membrane or matrix, with each site showing distinct properties (Wong et al., 2017). For example, superoxide generated in complex I is released into mitochondrial matrix, whereas superoxide originating from complex III can be deposited into either the mitochondrial matrix or intermembrane. Theoretically, mtROS from complex III, could be a convenient signaling molecule which can easily cross the mitochondrial membrane (Wong et al., 2017). The conversion of superoxide to hydrogen peroxide by the mitochondrial antioxidant manganese superoxide dismutase (SOD2) facilitates shuttling from the mitochondrial matrix into the cytoplasm (Sena and Chandel, 2012). Mitochondrial hydrogen peroxide can be further reduced by the antioxidant systems peroxiredoxin (Prx) and glutathione peroxidase (Gpx) in the presence of a reducing equivalent of NADPH (Ribas et al., 2014). The alleviation of mitochondrial oxidative stress through the intraventricular administration of Prx3 or Prx3/Trx2 protects against cerebral ischemic damage, whereas a deficiency of the antioxidant system triggered by the genetic deletion of SOD2 has adverse effects (Murakami et al., 1998; Kim et al., 2002; Hwang et al., 2010).

mtROS accumulation may cause mitochondrial dysfunction in response to cerebral I/R injury. Mitochondrial respiration is inhibited early after cerebral ischemia, which can result in electron leakage due to mtROS generation (Galkin, 2019). It has further been reported that ischemia induces a rapid drop in mitochondrial complex I activity, potentially due to a structural conformational transition when complex I does not oxidize NADH or pump protons outside the matrix (Chouchani et al., 2016; Kahl et al., 2018). Following reperfusion, electron transfer in complex I is directed in the opposite manner to reduce oxygen and generate extensive mtROS (Chouchani et al., 2014; Chouchani et al., 2016). Mitochondrial Ca^2+^ is thought to be another trigger for mtROS accumulation, with the hypothesis in cerebral ischemia stemming from the observation that mitochondrial Ca^2+^ overload triggers the opening of the mPTP, possibly resulting in ROS generation and cessation of ATP synthesis (Gorlach et al., 2015). However, few studies have directly examined the relationship between mitochondrial Ca^2+^ overload and ROS production during cerebral I/R injury. Additionally, SOD2 activity decreases in response to cerebral ischemia due to protein acetylation regulated by Sirt3 (Klimova et al., 2020).

Owing to the high reactivity and toxicity of ROS, increased mtROS production in the setting of cerebral I/R could adversely affect mitochondrial dysfunction and finally impair the cerebral neuronal system. A recent study reported that mtROS induces Ca^2+^ release from the endoplasmic reticulum (ER) into the mitochondria, resulting in the collapse of the mitochondrial membrane potential, increased mtROS production, and increased parthanatos levels in the setting of cerebral I/R (Zhong et al., 2018). Moreover, mtROS could react with lipid to generate phospholipid hydroperoxides (PLOOHs) in an iron-dependent manner, which executes a nonapoptotic cell death, called ferroptosis (Gao M. et al., 2019; Jiang et al., 2021). Although many studies reported that inhibition of ferroptosis had a protective role following cerebral I/R injury (Wang P. et al., 2021; Hu et al., 2022; Li M. et al., 2022; Li T. et al., 2023), few studies provided direct evidence between mtROS and ferroptosis in cerebral I/R injury. However, the disorder of mitochondrial amino acid metabolism and respiratory rate appears to be implicated in the generation of mtROS and subsequent ferroptosis (Gao et al., 2015; Gao M. et al., 2019; Li T. et al., 2023). The removal of mtROS using MitoQ was postulated to inhibit inflammation via Sirt6 after cerebral ischemia. This finding confirms that mtROS play a critical role in the inflammatory response, although the underlying mechanism remains unclear (Peng et al., 2020). mtROS are also considered critical contributors to brain damage after reperfusion (Chouchani et al., 2016). In line with this notion, several studies have shown that the free radical scavenger NXY-059 could largely reduce infarct volume at the onset of reperfusion in animal models, supporting the idea that oxidative stress is an important early driver of cerebral reperfusion (Marshall et al., 2001; Sydserff et al., 2002). However, the clinical outcomes of targeting ROS scavenging in stroke patients are always poor, possibly due to the half-life of antioxidants and the specificity of the human blood-brain barrier (Shuaib et al., 2007; Hong et al., 2022). Recently, nanomaterials have been utilized to facilitate the delivery of antioxidants and perform medical therapy, and this strategy is promising for the treatment of ischemic stroke in humans (Song et al., 2021; Wang Z. et al., 2023).

## 6 Dysregulation of mitochondrial turnover in cerebral I/R injury

A continuous turnover process involving the generation of new mitochondria and removal of senescent and damaged mitochondria constitutively occurs within cells to maintain a certain population of healthy mitochondria. Indeed, several studies have shown that cells can possess damaged mitochondria but remain viable, undergoing a sequential event of mitochondrial biogenesis, mitophagy together with fission/fusion, and ultimately repopulation with functional mitochondria (Suliman and Piantadosi, 2016). The mitochondrial proteome comprises approximately 1,100–1,500 proteins, including imported proteins encoded by nuclear genes, and 13 proteins encoded by mtDNA. The transcriptional control of these genes during mitochondrial biogenesis is required for mitochondrial rejuvenation (Hock and Kralli, 2009). A major breakpoint in understanding how these different gene subsets are fine-tuned was the identification of Peroxisome-proliferator-activated γ coactivator-1α (PGC-1α) as a transcriptional integrator in mitochondria (Wu et al., 1999; Scarpulla, 2008). Subsequent research revealed that PGC-1α (acting as a transcriptional coactivator of mitochondrial transcription factor A [TFAM]), nuclear respiratory factor 1 (NRF-1), and nuclear respiratory factor 2 (NRF-2) together potentiate mitochondrial biogenesis (Wu et al., 1999; Scarpulla, 2008). Findings in animal models further suggested that the loss of mtDNA occurs in the cerebral ischemic phase, and that this decrease could be fully recovered within a short period of ischemia following reperfusion (Chen et al., 2001; Shi et al., 2022). In addition, enhance mitochondrial biogenesis through the upregulation of TFAM or inhibition of glycogen synthase kinase-3 (GSK-3) was found to mitigate neuronal death and cerebral injury following ischemic insult (Hokari et al., 2010; Valerio et al., 2011). Little is known about the expression of these genes during ischemia; however, their expression levels have been shown to be elevated following reperfusion *in vivo* (Li et al., 2016). Taken together, these findings suggest that mitochondrial biogenesis plays a beneficial role in the replacement of dysfunctional mitochondria in neurons. To protect cells or subcells from stress conditions, cells have a quality-control system that sweeps up harmful cellular components, including individual proteins, vesicles, and damaged organelles. Given that neuronal mitochondria are active and can produce more ROS, these mitochondrial proteins may be at a higher risk of damage in response to cerebral I/R injury and need to be degraded to refresh the mitochondria (Goldberg, 2003; Misgeld and Schwarz, 2017). However, few studies have investigated the degradation of individual mitochondrial proteins in neurons. LON is a soluble ATP-dependent protease with proteolytic activity in the mitochondrial matrix (Desautels and Goldberg, 1982). Indeed, one prior study showed that both transcriptional and protein expression of the mitochondrial ATP-dependent protease Lon were downregulated after brain ischemic insult in rats (Hori et al., 2002). Another mitochondrial protease named serine protease HtrA2/Omi sites in the intermembrane, and could translocate into the cytosol during apoptosis. HtrA2/Omi, a mediator of apoptosis, is upregulated in response to cerebral and myocardial I/R. Inhibition of HtrA2/Omi activity using pharmaceutical drug prevented against neuronal (myocardial) apoptosis and cerebral (myocardial) infarction (Liu H. R. et al., 2005; Althaus et al., 2007). In contrast, HtrA2/Omi mutant (Ser276Cys) mice develop neurodegenerative diseases due to progressive mitochondrial dysfunction and ultimately juvenile death (Jones et al., 2003). In support of this notion, one study reported that the proteolytic activity of HtrA2/Omi plays an important role in preserving healthy mitochondria in the mouse brain cortex, while the loss of proteolytic activity results in increased cerebral I/R injury (Meng et al., 2022). Taken together, these findings indicate that the primary function of HtrA2/Omi is to digest damaged mitochondrial proteins, ensure mitochondrial health, and induce apoptosis under various stress (Vaux and Silke, 2003). To remove large assemblies of dysfunctional mitochondrial components, such as nucleic acids, proteins, lipids, and fragmented protein pieces, the mitochondrial membrane(s) wrap around these constituents to form mitochondria-derived vesicles (MDVs) (Misgeld and Schwarz, 2017; Chaiyarit and Thongboonkerd, 2023). These MDVs subsequently bud off from mitochondria and are delivered to different destinations such as peroxisomes, lysosomes, or multivesicular bodies, or are exocytosed (Misgeld and Schwarz, 2017). However, the significance of MDV-mediated mitochondrial quality control in cerebral I/R injury is not well established, and remains to be explored.

Mitophagy is a degradative pathway with the potential to eliminate fragmented or whole mitochondria. Mitophagy can proceed via two pathways: ubiquitin-dependent and ubiquitin-independent (Harper et al., 2018). Ubiquitin-dependent mitophagy is driven by the ubiquitin ligase parkin and its activating kinase, PINK1. In addition to ubiquitin-dependent mitophagy, mitophagy can be initiated by different mitophagy receptors in mammals including NIX, BNIP3 and FUNDC1 (Novak et al., 2010; Quinsay et al., 2010; Hanna et al., 2012; Liu et al., 2012). Two groups have suggested that mitophagy is activated following an I/R insult (Zhang X. et al., 2013; Lan et al., 2018), and ischemia alone results in neuronal mitophagy deficiency but not autophagy activation (Zhang X. et al., 2013; Wu X. et al., 2021). In addition, several studies have proposed that enhancing mitophagy could exert a protective effect against cerebral ischemia or I/R injury (Li et al., 2014; Chen et al., 2015; Di et al., 2015; Shen et al., 2017; Cai et al., 2021; Wu X. et al., 2021). These findings demonstrate that the selective removal of damaged mitochondria by mitophagy is critical for preserving neuronal function and brain infarction area during cerebral I/R injury. Interestingly, the inhibition of autophagy activation has also been demonstrated to play a protective role in neuronal survival and the progression of cerebral infarction after ischemic insult, which is accompanied by an improvement in mitochondrial function (Wen et al., 2008; Baek et al., 2014). To date, the extent to which mitophagy occurs during cerebral ischemia or I/R remains uncertain. However, should be noted that the activation of Parkin/PINK1-dependent mitophagy relies heavily on ATP, and cerebral neurons are unlikely to have enough ATP to support the activation after long-term ischemic exposure (Harper et al., 2018); (2) PINK1/Parkin pathway to mitophagy is largely cut off if all the mitochondria in neurons are poisoned (Van Laar et al., 2011; Cai et al., 2012); (3) The functions of Parkin and PINK1 are not limited to initiating mitophagy (Johnson et al., 2012; Muller-Rischart et al., 2013; Morais et al., 2014). Therefore, more detailed investigations of Parkin/PINK1-dependent mitophagy are required.

Mitochondria are highly dynamic, undergoing constant fission and fusion, and are capable of separating damaged mitochondria from healthy parts or functionally repairing damaged mitochondria by diluting accumulated mutational mtDNA and oxidized proteins, respectively (Twig et al., 2008; Youle and van der Bliek, 2012). The mitochondrial fission process is primarily mediated by dynamin-related protein 1 (Drp1), while three different GTPases, mitofusin (MFN) 1/2, and optic atrophy protein 1 (Opa1), oversee this fusion (Chen et al., 2023). The special roles of mitochondrial dynamics, particularly fission, are closely related to mitophagy in cerebral I/R injury (Burman et al., 2017; Zhang and Yu, 2018). During cerebral I/R, the mitochondrial dynamic balance is disturbed, accompanied by fission activation and fusion inhibition (Kumari et al., 2012; Zeng et al., 2022; Zhao et al., 2022). Genetic or pharmacological inhibition of Drp1-dependent mitochondrial fission protects the brain from ischemia and I/R injury (Grohm et al., 2012; Zhang N. et al., 2013; Zhao et al., 2014; Ma et al., 2016; Flippo et al., 2018; Flippo et al., 2020; Ali et al., 2022; Zhao et al., 2022). However, in to the protective roles of Drp1 in ischemia and I/R injury, many other pathways mediated by Drp1 have also been identified, including apoptosis/cell death, inflammation, oxidative stress, and opening of the mPTP (Grohm et al., 2012; Zhang N. et al., 2013; Zhao et al., 2014; Ma et al., 2016; Flippo et al., 2018; Zhao et al., 2022). Thus, Drp1 is considered a potential target for the treatment of cerebral ischemia and I/R injury. Although several studies have suggested that mitochondrial fusion enhancement is associated with the protection of cerebral ischemia or I/R injury, direct examination of the roles of mitochondrial fusion in cerebral ischemia or I/R is sparse (Zhang and Yu, 2018; Gao M. et al., 2019; Huang S. et al., 2023; Xu et al., 2023; Gao J. et al., 2019; Huang Q. et al., 2023).

## 7 Therapeutic implications

Mitochondrial function has been considered as an important therapeutic target for treatment cerebral I/R injury. As discussed above, it is well established that mitochondrial impairment was coupled with cerebral I/R injury. Therapeutical targeting of dysfunctional mitochondria may give a promising direction and yield an effective treatment. However, prior efforts targeted to mitochondrial permeability transition pore, monoamines metabolism, oxidative stress in cerebral I/R injury, have a limited achievement though promising data acquired from preclinical studies (Nighoghossian et al., 2015; Ramezani et al., 2020; NCT02530307). As new discoveries of mitochondrial function in cerebral I/R injury are increasing, rational design of novel therapies holds promise. Studies showed that damaged tissues caused by ischemia can be reversed after infusing healthy mitochondria (Weixler et al., 2021; Doulamis et al., 2020; Orfany et al., 2020). An ongoing clinical study that aimed to transplant healthy autologous mitochondria into cerebral vessels for treatment cerebral ischemia in patients was conducted in USA (NCT04998357, clinicaltrials.gov), which may provide us an encouraging finding.

Butylphthalide was showed to preserve mitochondrial function in several ways, including normalization of mitochondrial oxidative stress, inhibition of mitochondrial Ca^2+^ overload, mitochondrial apoptosis and inflammation (Chen et al., 2019; Que et al., 2021; Li T. et al., 2023; Huang S. et al., 2023). Butylphthalide treatment was observed to increase regional cerebral blood flow, reconstruct microcirculation at the ischemic area (Guo et al., 2023; Zhang et al., 2020). Patients with ischemic stroke who received a 90-day treatment with butylphthalide had a favorable functional outcome compared with placebo (Wang A. et al., 2023). However, the risk of long-term death or dependence requires further evaluation.

Sovateltide as a neural progenitor cell therapeutic agent, exerts the ability to improve neurological and motor functions, and subsequently reduces infarct volume and oxidative stress damage after cerebral ischemic injury in animals (Leonard et al., 2011; Leonard et al., 2012). Several ongoing and completed clinical studies were conducted to test the safety and efficacy in patients with acute cerebral ischemic stroke in India (NCT05955326; NCT05691244; NCT04047563). Sovateltide was shown to improve mitochondrial morphology and biogenesis in ischemic brain after stroke, and thus mediated neural regeneration and repair (Ranjan et al., 2020). By now, sovateltide was approved for the treatment of cerebral ischemic stroke within 24 h of stroke onset in India, 2023.

Metformin was first used as anti-diabetic drug because of the ability to reduce serum glucose levels. However, accumulating data provided evidence that metformin is also beneficial for other diseases, such as cancers, cardiovascular diseases and aging. Metformin is capable to inhibit mitochondrial complex I and activated the AMPK (AMP activated protein kinase) pathway (Cameron et al., 2018; Zhou et al., 2001). In support of this notion, pre-stroke metformin use in diabetic patients is coupled with improved stroke prognosis (Westphal et al., 2020; Chien et al., 2017; Kersten et al., 2022), which could be ascribed to AMPK activation (Wang M. et al., 2023). Along the lines of glucose metabolism, sodium-glucose cotransporter-2 (SGLT2) inhibitor, another anti-diabetic drug, that could prevent glucose reabsorption in the kidney, was shown to low stroke risk in patients with diabetes and atrial fibrillation (Chang et al., 2023). Interestingly, SGLT2 inhibitor has no effect on ischemic stroke in patients with type 2 diabetes (Zhou et al., 2019). Furthermore, a combination clinical therapy by using pioglitazone and SGLT2 inhibitor for the treatment of recurrent stroke in diabetic patients was completed, but the official results have not yet been published (NCT04419337).

## 8 Conclusion and perspective

Mitochondria supply the majority of ATP via OXPHOS, a complex system that coordinates mitochondrial metabolism to sustain cellular physiological functions. During ATP synthesis, mtROS byproducts are primarily derived from the mitochondrial ETC, which can either serve as molecular signals or cause apoptosis and cell death. Furthermore, mtROS can damage mtDNA and proteins, which can escape the mitochondria and have the potential to act as molecular signals. As such, it is important to ensure a healthy mitochondrial state that can be controlled by mitochondrial biogenesis (mitochondrial quality control) and a balance between mitochondrial fission and fusion. Accumulated damaged mitochondria can be removed by mitophagy or programmed death. Mitochondria also act as sensors and buffers that circulate Ca^2+^ between mitochondria and other cellular compartments, particularly the sarcoplasmic reticulum (SR). Herein, we have listed some mitochondrial output signals in the setting of cerebral ischemia or I/R injury without discussing the mitochondria receiving or sensing incoming signals. However, it is impossible to paint a complete picture of mitochondrial output signals during cerebral ischemia or I/R. Our goal in presenting this review was to facilitate a better understanding of the role of mitochondria in cerebral ischemia or I/R injury. Mitochondrial function is not only confined to mitochondrial energetics, but also acts as a network that regulates cellular physiological or pathological mechanisms. We have only begun to understand the reciprocal relationship between mitochondrial output signals and the onset and development of ischemic stroke. As listed above, multiple mitochondrial dysfunctional processes are involved in cerebral I/R injuries, and various drugs targeting to mitochondria were evaluated in clinic but received a limited beneficial effect. Therefore, combination of drugs and interventional therapies are likely to be the optimum therapeutic strategy in ischemic treatment. Another major challenge is the yawning gap between preclinical and clinical studies. Previously, therapeutic drugs for ischemic stroke treatment are always not delivered to the brain due to the presence of blood–brain barrier (BBB). The development of nanomedicine for brain delivery might be the key to improve ischemic stroke by targeting mitochondrial function (Li X. et al., 2022; Wang M. et al., 2023). Thus, the discovery of novel molecular mechanisms in mitochondrion and the development of advanced engineer biotechnology pose promising opportunities for future clinical trials to cure ischemic stroke.
